# Leaf water dynamics of *Arabidopsis thaliana* monitored *in-vivo* using terahertz time-domain spectroscopy

**DOI:** 10.1038/srep02910

**Published:** 2013-10-09

**Authors:** E. Castro-Camus, M. Palomar, A. A. Covarrubias

**Affiliations:** 1 Centro de Investigaciones en Optica A.C., Loma del Bosque 115, Lomas del Campestre, Leon, Guanajuato 37150, Mexico; 2 Depto. Biologia Molecular de Plantas, Instituto de Biotecnologia, Universidad Nacional Autonoma de Mexico, A.P. 510-3. Cuernavaca, Morelos, Mexico 62250

## Abstract

The declining water availability for agriculture is becoming problematic for many countries. Therefore the study of plants under water restriction is acquiring extraordinary importance. Botanists currently follow the dehydration of plants comparing the fresh and dry weight of excised organs, or measuring their osmotic or water potentials; these are destructive methods inappropriate for *in-vivo* determination of plants' hydration dynamics. Water is opaque in the terahertz band, while dehydrated biological tissues are partially transparent. We used terahertz spectroscopy to study the water dynamics of *Arabidopsis thaliana* by comparing the dehydration kinetics of leaves from plants under well-irrigated and water deficit conditions. We also present measurements of the effect of dark-light cycles and abscisic acid on its water dynamics. The measurements we present provide a new perspective on the water dynamics of plants under different external stimuli and confirm that terahertz can be an excellent non-contact probe of in-vivo tissue hydration.

Terahertz time-domain spectroscopy (THz-TDS) is a technique developed over the last 30 years^1^,^2^. Although expensive, this technology is now commercially available and is starting to find applications within various fields^3^,^4^ including chemistry and biology^5^,^6^. Most dehydrated biological tissues are transparent (or at least partially transparent) in this spectral region, while water is highly opaque in this band^7^; therefore, terahertz is an excellent non-contact probe of water content in biological tissues as schematically depicted in Fig. 1b^8^. Additionally, time-domain spectroscopy has the advantage of operating in the microwatt power regime with extraordinary signal to noise performance^9^; therefore, the extremely low radiation intensities used to probe the hydration state of leaves are expected to produce negligible heating effects on the tissue under examination. The terahertz spectral band refers to radiation with frequencies between 100 GHz and 3 THz (wavelength between 100 μm and 3 mm). Because of these advantages, in recent years, microwave (i.e. sub-THz)^10^ and THz^11^,^12^ radiation has been used as a non-contact probe of water content in plant tissues. In addition, water distribution in plant tissue has been imaged using THz radiation^13^,^14^.

Although useful and informative, most of the methods commonly used to measure water content in plant tissues, such as water potential or relative water content, imply damage or separation of the organs of interest from the plant. Such procedures do not allow following changes of water content in real-time during short time periods upon particular stimuli.

In this article, we present a series of measurements of the *in-planta* water dynamics of *Arabidopsis thaliana* under various stimuli. In particular we followed the water loss of cauline leaves under water restriction in plants grown in two substrates with different water retention capacity. This was achieved by probing water content with broad band terahertz pulses. Similar measurements were done in plants under optimal irrigation. We conclude that the substrate water retention capacity plays an important role in the water content dynamics of the plant. In addition, we used this technique to detect the water content changes produced by stomatal closing during day/night cycles as well as induced by abscisic acid (ABA) treatments.

## Results

**Figure 1:**
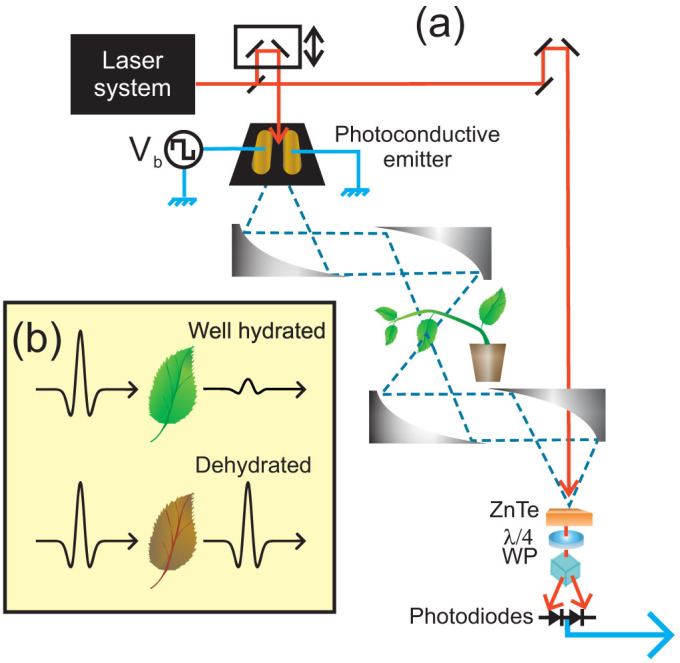
(a) Schematic representation of the terahertz time-domain spectrometer used to monitor the real-time leaf water content. Ultrashort laser pulses are converted into terahertz pulses using a photoconductive emitter. These pulses are collected from the emitter and refocused onto the leaf under study using off-axis parabolic mirrors. The terahertz radiation transmitted through the leaf is collected and refocused onto the ZnTe detector crystal by two additional parabolic mirrors. (b) Schematic representation of the principle of operation, by which THz measurements are performed for a hydrated and dehydrated leaf. Given that water is highly absorptive in the terahertz band, well-hydrated tissues (top) will attenuate the transmitted THz pulses more than dehydrated tissues.

The terahertz transmission experiment is depicted in Fig. 1a. In brief, terahertz radiation is produced, transmitted through the leaf under study and detected after. The degree of attenuation of the radiation in the leaf can be related to the amount of water present in the tissue (Fig. 1b). By using an effective medium theory model^15^ it is possible to determine the weight percentage of water in the leaf (ie. what percentage of the total weight of the leaf is actually water), this is denoted as *Wt. %* from now on. Further details concerning the terahertz measurement method and data analysis are presented in the Methods section.

### Detection of changes in leaf water content during water restriction

**Figure 2:**
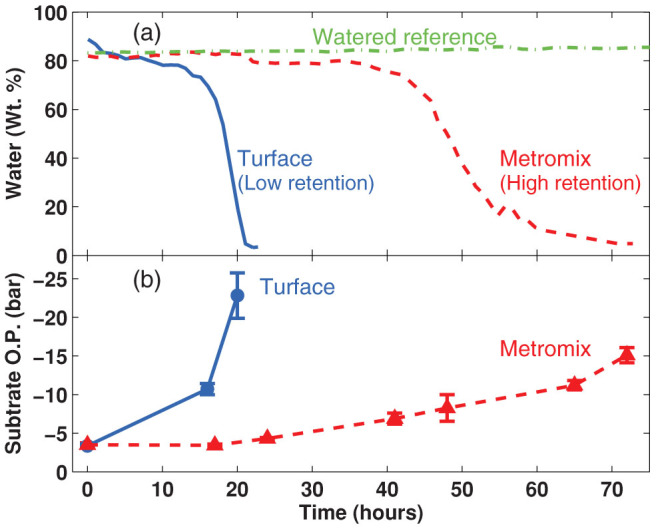
(a) Comparison of the water content (water weight percentage) from caulinar leaves of Arabidopsis plants grown in two substrates with different water retention capacity: Metromix (higher retention, dashed line) and Turface (lower retention, continuous line). Irrigation was halted at t = 0. Leaf tissues from plants grown in Turface show faster water loss rate compared to plants grown in Metromix. Plants in Turface showed relatively slow dehydration over the first 8 to 10 h with a sudden rate change after approximately 15 hours of water supply halt, dehydration of the tissue became very rapid and tissues lost most of their water in the following 5 hours. Samples from Metromix showed a relatively steady tissue water content over the first 30 h and slower dehydration through the following 25 h, culminating with almost total water loss around 70 h after halting water supply. A continuously watered plant grown on Metromix substrate was also monitored as reference (dash-dotted line). (b) The osmotic potential of both substrates was also determined. This shows the difference in water retention capacity of these substrates.

After mounting a cauline leaf, as shown in Fig. 1a, the spectrometer was programmed to acquire a terahertz waveform every hour. As shown in Fig. 2a, the water content (weight percentage) from these two recordings indicate a clear difference in the water loss rate over a period of about 70 h, between cauline leaves from plants grown in Turface and those from plants grown in Metromix. As expected, the water loss rate was faster during water deprivation in leaves from plants maintained in a low water retention substrate. In both cases water loss seems to occur in three-phases. A first slow phase lasting 12 h with a water loss rate of −1.3% h^−1^ for plants grown in Turface, whereas for plants grown in Metromix lasted approximately 40 h with a rate of −0.2% h^−1^. Then a second phase showing a faster water loss in both conditions, presenting a water loss rate of −14.6% h^−1^ for leaves from plants kept in Turface and −3.5% h^−1^ for those maintained in Metromix. After approximately 9 h for plants in Turface and 15 h for plants in Metromix, leaf water content was reduced to about 4% and further changes were not significant (Fig. 2a). In order to demonstrate the difference of water retention capacity of the two substrates, the osmotic potential of both soil types was measured as function of time after water supply was halted. In Fig. 2b the time dependent osmotic potentials are shown. Turface showed a rapid dehydration consistent with a significant decrease in osmotic potential over the first 20 hours. In comparison, Metromix showed a slower dehydration, in agreement with the observed reduction in its osmotic potential.

### Detection of changes in leaf water content during dark-light periods and ABA treatment

**Figure 3:**
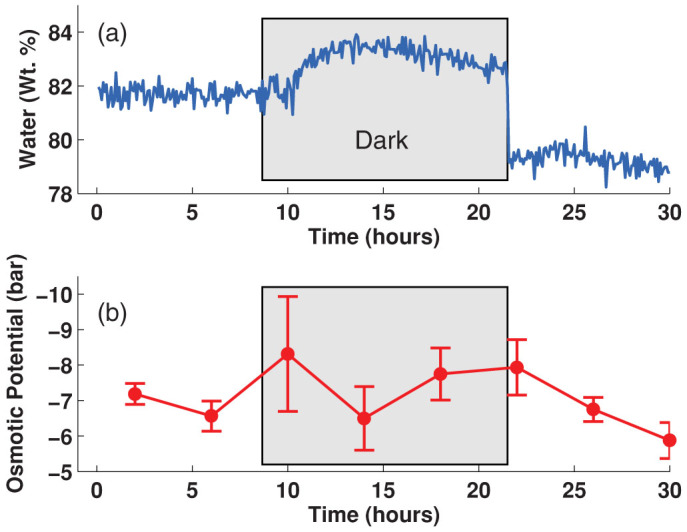
Changes in water content and osmotic potential in leaf tissues of Arabidopsis plants grown in Metromix during water loss, comparing day and night periods (a) Real-time water weight fraction recorded at 5 min intervals during the indicated time. (b) Osmotic potential of Arabidopsis leaves, values determined from a parallel experiment as the one described in (a). (Bars indicate standard error).

We also detected a significant change in leaf water content between light and dark periods when following the dehydration process. The spectrometer was setup to acquire a terahertz waveform every 5 minutes in order to analyze this phenomenon in more detail. As shown in Fig. 3a, the water content increased over the first 3 h of darkness, and it stayed steady until a light period started again. These real-time measurements of leaf water content correlate very well with stomatal conductance measurements reported for light and dark periods^16^. We also performed a series of measurements of osmotic potential of cauline leaves from plants subjected to identical growth and treatment conditions (Fig. 3b), which show rather large uncertainties but correlate also consistently with the terahertz measurement. This indicates that changes in the average absorption coefficient reflect variations in the leaf tissue water content caused by the closing and opening of the stomata induced by dark and light periods, respectively as reported in Ref. 17.

**Figure 4:**
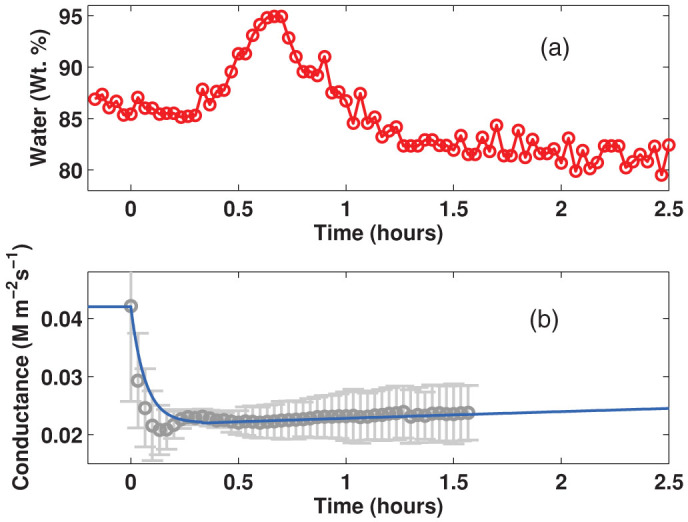
(a) Effect of ABA application on the water content of Arabidopsis cauline leaves. Water weight percentage determined at 2 min intervals reveals transitory leaf water content changes upon the stomatal closing induced by the ABA treatment (at t = 0). (b) Stomatal conductance of Arabidopsis leaves after application of ABA. The gray dots are measured values with their respective uncertainties (Bars indicate standard error), the continuous curve is an approximation of conductance time dependence by a double exponential.

In order to verify the ability of this THz method to detect water content differences caused by stomatal aperture/closure, the effect of ABA treatment was tested. The THz spectrometer was programmed to acquire a terahertz waveform every 2 minutes to detect variations in the water content due to stomata closure induced by the ABA treatment. Figure 4a shows that a transitory small but detectable increase in water content occurred just after the leaf was sprayed with ABA solution (at t = 0). The increase of water content, attributed to the stomatal closure induced by ABA is consistent with the light/dark observation. In addition the water conductance of Arabidopsis leaves was measured as function of time after application of ABA solution. The evolution of the stomatal conductance is presented in Fig. 4b, which shows a sudden drop, caused by the stomatal closure in the following minutes, and a slow recovery over the following hours. These data can be fitted by two exponentials with time constants 4 min and 969 min as depicted in the figure by a continuous line.

As can be appreciated in Fig. 4, THz measurements can detect a significant increase in leaf water content upon ABA treatment, followed by a rapid decrease. Simultaneously, the stomatal conductance reflects the effect of the ABA treatment, by a rapid drop and a subsequent slow increase in stomatal conductance, these two observations although consistent in trend show different timescales. These results are consisten with the fact that the THz radiation is sensitive to water content variations in the leaf that are not only caused by the stomatal closing induced by ABA but also those changes resulting from the stomatal aperture adjustment and from the leave's intake (or loss) of water by other mechanisms such as the dehydration of the substrate as shown in the first experiment presented in this article, therefore it is not expected that the timescales of both measurements should be identical.

## Discussion

The strong attenuation of terahertz radiation by water makes radiation in this spectral band a highly sensitive non-contact probe of hydration in plants as well as other materials^18^. In addition, a good number of dielectric materials, such as dehydrated biological tissue, are mostly transparent in this spectral region allowing us to determine the water content of leaves from their THz transmission. This technique offers significant advantages when compared to the traditional methods, such as pressure chambers or psychrometry, used in plant science to estimate water content in plant tissues. Unlike such procedures, THz spectroscopy is non-destructive, does not require contact with the sample, and allows continuous monitoring of leaf water content that could be affected by *in vivo* water uptake or water loss processes, therefore giving unprecedented capability to follow the plant water dynamics.

In this work, we tested the suitability of this technology to measure the changes of water content of leaves from Arabidopsis plants under optimal and suboptimal watering conditions, in two different substrates. The application of this technique to the analysis of the water content dynamics in cauline Arabidopsis leaves when plants are subjected to low water availability allowed us to follow the real-time water loss kinetics. The continuous registration of the data allowed distinguishing three stages or phases with different water loss rates during water restriction on both substrates, showing that even during the same treatment plants are facing periods with considerably different stress intensity which could lead to a different set of plant responses. The possibility to perform this kind of measurements allows a much more detailed monitoring of the water content in tissues, leading to a better interpretation of physiological, cellular or molecular data from particular experiments. In addition, we showed that this technique is sensitive enough to detect the changes in leaf tissue water content produced by the closing and opening of stomata induced by dark/light periods or by ABA treatment. The comparison between the leaf water content obtained by osmotic potential measurements and that obtained by the uninterrupted recording of THz transmission showed that this last procedure allowed a more sensitive and precise determination of the changes in water content during this process.

Currently THz-TDS is still a relatively expensive technology, as of the time of this publication TDS system list prices start on the order of one hundred thousand US dollars, and therefore the access to this kind of equipment is still limited. Yet the recent and rapid development of THz systems for industrial and research purposes opens real possibilities of this costs to drop significantly in the coming years which will at its time allow their implementation in plant biology laboratories^19^,^20^. In addition to dynamics, the development of a method that allows imaging water content in leaves and other tissues has been demonstrated^21^,^22^. The measurements presented here provide a new perspective on the water dynamics of Arabidopsis, in particular, and of plants in general. It is expected that the terahertz technique will provide unprecedented insights in the water dynamics of plants in the future. The development of compact, mobile and more cost effective experimental setups will enable them to be used in the search for selection markers in crop drought improvement programs.

## Methods

### Sample preparation

Wild type *Arabidopsis thaliana* (Landsberg erecta ecotype) (At) were grown on Petri dishes with solid MS 1X pH 5.7 (4.3 g/L Murashige and Skoog salts, 1% sucrose, 0.5 g/L MES, 0.8% agar) in a growth chamber at a controlled temperature of 21°C, 16/8 hrs light/dark photoperiod, under 80–100 μmol m^−2^ s^−1^ (~18 W m^−2^) of light intensity and a relative humidity of 60–70% for two weeks. Seedlings were transplanted to pots (76.2 mm × 73.0 mm × 54.0 mm) containing substrates of Metro-Mix 200 or Turface MVP (Hummert International Earth City, MO USA), the latter one being the substrate with lower water retention. Plants were grown under the conditions described above and watered with nutritive solution (H_3_BO_3_ 70 mM, CuSO_4_ 0.5 mM, NaMoO_4_ 2 mM, CoCl_2_ 0.01 mM, MnCl_2_ 14 mM, ZnSO_4_ 1 mM, NaCl 10 mM) and sterile water (every other time). When plants reached their reproductive stage (approximately after 4–5 weeks) they were exposed to progressive water loss by interrupting water supply (Time = 0). At this point, a potted plant was transferred to the THz-TDS setup for analysis, where it was maintained over the duration of the experiments at 21.5°C and 20–25% relative humidity, with a 12/12 Hr light/dark photoperiod. Because of their accessibility, terahertz pulses were propagated through *in planta* cauline leaves.

### Determination of osmotic potential

Osmotic potential of cauline leaves was determined during the water loss treatment described above from plants grown in Turface MVP or Metro-Mix 200 (Hummert International, Earth City, MO USA). One cauline leave from a treated and one from a well watered plant was collected every 4 hours and immediately frozen in liquid nitrogen and subjected to five cycles of freezing (1 min in N_2_ liquid), thawed (10 min at 25°C) and then centrifuged 20 min at 14000 rpm in an Eppendorf centrifuge. One microliter of the supernatant was collected and diluted in 9 μl of sterile water. The osmolality of the sample was determined in a Vapor Pressure Osmometer (Wescor VAPRO Model 5600, Wescor, Inc., Logan UT USA) following the instructions of the manufacturer. The values obtained in mmol/Kg were converted to bars considering 1 OsM/Kg = −25 bar^23^.

### Determination of stomatal conductance

Stomatal conductance was measured with a Li-Cor LI-6400 apparatus following manufacturer's instructions (Li-Cor, Lincoln, NE, USA). During measurements, plants were maintained under similar environmental conditions as those present during THz evaluations. The cell (6 cm^2^) parameters were as follows: 25°C, 200 μmol photons m^−2^ s^−1^ and an air flow 300 μL s^−1^. Environmental humidity was approximately 45%, slightly higher given the weather conditions at the time of the determinations.

### ABA treatment

Abscisic acid (ABA) solution (100 μM, prepared in ethanol) was sprayed on both sides of cauline leaves from Arabidopsis plants grown in Metro-Mix 200 under optimal conditions. This treatment was applied 15 min after the start of THz data acquisition.

### Terahertz spectroscopy

**Figure 5:**
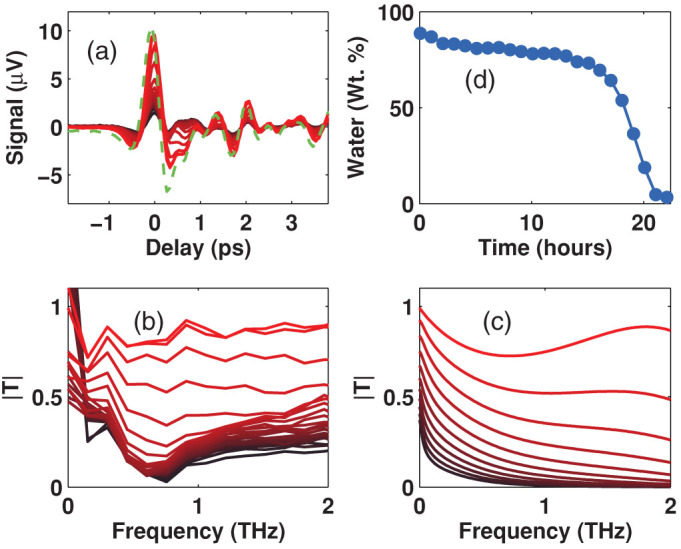
(a) THz time-domain data set taken over a 23 h period. Measurements were taken from cauline leaves from Arabidopsis plants grown in Turface as described in Material and methods. Water supply was halted at time zero and THz pulses were recorded every hour. Each red line represents a measurement from each hour. The transmitted THz pulse measured at t = 0 (darker red) is small and increase in amplitude upon plant dehydration as time passes (brighter red). The dashed (green) line corresponds to the reference pulse measured in the absence of sample. (b) Calculated transmission as function of frequency determined from the reference pulse and the pulses measured each hour (each line) showing that transmittance was low at early times (dark red lines), and increased over time (bright red lines). (c) Theoretical transmission calculated by effective medium theory for water weight fractions from 0% (dark red lines) to 100% (bright red lines). (d) The water weight fraction present in the leaf as a function of time after restriction of water supply.

A terahertz time-domain spectrometer (Fig. 5a) was built based on a Ti:Sapphire oscillator producing pulses of light with a duration of approximately 33 fs at 80 MHz repetition rate, with an average power of 200 mW. About 70% of the laser power was sent to a computer controlled delay line and subsequently used to produce terahertz pulses in a semi-insulating GaAs photoconductive emitter biased with a 100 V square wave at 12 KHz across a 400 mm gap. The terahertz radiation was collected by an off-axis parabolic mirror and refocused onto a cauline leaf. The radiation transmitted through the sample was then collected and refocused on a 1 mm thick [110] ZnTe crystal by two additional parabolic mirrors. The remaining 30% of the laser pulse was sent through a separate path to the ZnTe crystal in order to perform electro-optic detection of the terahertz pulse.

### Signal processing

The hydration monitoring experiment consists in recording a terahertz pulse waveform *E_j_(t)* in the time domain (Fig. 5a) at regular time intervals. Each of these waveforms represents a “snapshot” of leaf water content at the times recorded. A reference pulse *E_ref_(t)* in the absence of leaf is also recorded (dashed line). A Fast Fourier Transform algorithm was used in order to obtain their amplitude and phase spectra as function of frequency *f*. The transmission spectra for each measurement are subsequently calculated as described in Ref. [24]. The amplitude of the transmission coefficient function for each measurement is shown in Fig. 5b. In order to determine the amount of water present in the leaf, effective medium theory can be used to theoretically calculate the transmission for any water fraction present in the tissue^15^, the calculated transmission spectra for water fractions from 0 to 100% (in 10% steps) are presented in Fig. 5c. A computer program was used to adjust the water fraction in the theoretical model to find the best fit between the theoretical and each experimental (in Fig. 5b) transmission spectra in order to determine the water content at each time. The resulting water fractions are shown in Fig. 5d.

## Author Contributions

E.C.-C. conceived the study supported by A.A.C., M.P. grew the Arabidopsis samples and performed the osmotic potential and stomatal conductance experiments. E.C.-C. performed the spectroscopy experiments. E.C.-C. and A.A.C. wrote the paper. All authors reviewed the manuscript.

## Notes

**Reprints and permission** information is available online at http://npg.nature.com/reprintsandpermissions/.
